# Underlying Tremor Improvement with Consistent Use of Transcutaneous Afferent Patterned Stimulation in Patients with Essential Tremor

**DOI:** 10.5334/tohm.1091

**Published:** 2026-01-09

**Authors:** Stuart H. Isaacson, Elizabeth Peckham, Winona Tse, Melita T. Petrossian, Michael J. Soileau, Mark Lew, Cameron Dietiker, Nijee Luthra, Pinky Agarwal, Rohit Dhall, John Morgan, Ejaz A. Shamim, Holly A. Shill, Fernando L. Pagan, Pravin Khemani, Jessica Tate, Lan Luo, William Ondo, Mark Hallett, Chiahao Lu, Kathryn H. Rosenbluth, Scott L. Delp, Rajesh Pahwa

**Affiliations:** 1 Parkinson’s Disease and Movement Disorders Center of Boca Raton, Boca Raton, FL, USA; 2 Central Texas Neurology Consultants, Round Rock, TX, USA; 3 Department of Neurology, Icahn School of Medicine at Mount Sinai, New York, NY, USA; 4 Pacific Neuroscience Institute, Pacific Movement Disorders Center, Santa Monica, CA, USA; 5 Texas Movement Disorder Specialists, Georgetown, TX, USA; 6 Department of Neurology, Keck School of Medicine of USC, Los Angeles, CA, USA; 7 Department of Neurology, University of California San Francisco, San Francisco, CA, USA; 8 Booth Gardner Parkinson’s Center, Evergreen Health, Kirkland, WA, USA; 9 Movement Disorders Clinic, University of Arkansas for Medical Sciences, Little Rock, AR, USA; 10 Department of Neurology, Augusta University, Augusta, GA, USA; 11 Mid-Atlantic Kaiser Permanente Neuroscience Institute, Kaiser Permanente Mid-Atlantic States, Rockville, MD, USA; 12 Department of Neurology, Mid-Atlantic Permanente Research Institute, Kaiser Permanente Mid-Atlantic States, Rockville, MD, USA; 13 Barrow Neurological Institute, University of Arizona, Phoenix, AZ, USA; 14 Department of Neurology, Georgetown University Hospital, Washington, DC, USA; 15 Department of Neurology, Swedish Neuroscience Institute, Seattle, WA, USA; 16 Department of Neurology, Wake Forest School of Medicine, Winston-Salem, NC, USA; 17 Department of Neurology, Beth Israel Deaconess Medical Center, Harvard Medical School, Boston, MA, USA; 18 Department of Neurology, Houston Methodist Neurological Institute, Houston, TX, USA; 19 Department of Neurology, Weill Cornell Medical College, New York, NY, USA; 20 Human Motor Control Section, National Institute of Neurological Disorders and Stroke, Bethesda, MD, USA; 21 Cala Health, San Mateo, CA, USA; 22 Stanford University School of Medicine, Palo Alto, CA, USA; 23 Department of Bioengineering and Mechanical Engineering, Stanford University, Palo Alto, CA, USA; 24 Department of Neurology, University of Kansas Medical Center, Kansas City, KS, USA

**Keywords:** tremor, neuromodulation, transcutaneous afferent patterned stimulation, underlying tremor improvement

## Abstract

**Background:**

Transcutaneous afferent patterned stimulation (TAPS) is a non-invasive, wrist-worn neurostimulation therapy that has demonstrated acute and short-term lasting tremor reduction in patients with essential tremor (ET). However, the longer-term improvement in underlying tremor severity from consistent use of TAPS has not been fully explored.

**Methods:**

We conducted a retrospective analysis of the multicenter PROSPECT trial, which evaluated twice-daily TAPS use over three months in patients with ET. Underlying tremor improvement was assessed by comparing pre-stimulation tremor severity at baseline with pre-stimulation tremor severity at 1- and 3-month follow-up visits. Tremor severity was measured using the Bain & Findley Activities of Daily Living (BF-ADL) scale and the Tremor Research Group’s Essential Tremor Rating Assessment Scale (TETRAS). Responders were defined as patients demonstrating at least a 1-point improvement on any qualifying task.

**Results:**

Among 192 patients with available data, pre-stimulation BF-ADL scores improved significantly by 2.0 points at 1 month and 2.7 points at 3 months compared with baseline (p < 0.001). Pre-stimulation TETRAS scores also showed significant improvements at both time points (p < 0.001). Measurements at 1 and 3 months were made an average of 16.2 hours after the prior stimulation session. Over 80% of patients met responder criteria for underlying tremor improvement on BF-ADL and TETRAS at both follow-up visits. Improvements were observed even among patients using TAPS approximately once daily.

**Conclusions:**

Consistent use of TAPS was associated with significant improvement in underlying tremor severity in patients with essential tremor. These findings suggest that regular TAPS use may confer sustained therapeutic benefit.

Decades of bioengineering development, neurostimulation advances, and neuroscience research has led to the clinical development of non-invasive, individualized, wrist-based neurostimulation as a safe and effective therapy to reduce tremor that impairs daily activities in people living with essential tremor (ET). Non-invasive peripheral electrical neurostimulation has demonstrated safety and improved clinical outcomes in four randomized clinical trials (N = 23 [1], N = 77 [2], N = 276 [3], N = 125 [4]), two open-label studies (N = 205 [5], N = 17 [6]), analyses of real-world usage (N = 321 [7], N = 1,223 [8]), health care economic data (N = 459 [9]), and mechanistic studies [10,11].

Transcutaneous afferent patterned stimulation (TAPS) therapy is the most widely studied non-invasive neurostimulation therapy for ET, with more than 2,000 patients in published studies to-date [1,2,3,5,7,8,10,11,13,14,15]. The most widely used device cleared by the US Food and Drug Administration (FDA) for an indication of essential tremor delivers TAPS™ therapy (Cala Health). This device is also indicated to treat postural and kinetic tremors in Parkinson’s disease. TAPS therapy applies alternating bursts of stimulation pulses to the median and radial nerves at a frequency calibrated and individualized to each patient’s tremor frequency [1]. Although other non-invasive devices are emerging [16,17], the present analysis focuses on the duration of benefit of TAPS therapy.

Tremor improvement can be of shorter or longer duration; thus, it is important to define the terminology for measuring the duration of benefit with use of non-invasive peripheral neurostimulation. Pasqual-Valdunciel and colleagues recently defined “acute” effect as tremor improvement measured during a stimulation session, and “lasting” effect as tremor improvement measured in minutes or hours after a stimulation session [12]. In addition to the definitions of acute and lasting effects of tremor improvement, underlying tremor improvement can be evaluated after days, weeks, or months of consistent use of TAPS therapy. We define this as a change in the pre-stimulation tremor baseline, measured after a significant washout period to distinguish it from any short-term “lasting” effects. Measuring underlying tremor improvement provides valuable insights into whether consistent use of therapy yields a reduction in tremor severity. This concept complements acute and lasting effects and supports a more comprehensive framework for evaluating non-invasive neurostimulation.

In this report, we highlight findings from a retrospective analysis of the PROSPECT trial [5] (NCT03597100) on TAPS. The design of this study enabled analysis of acute and lasting benefit from as-needed use as well as underlying tremor improvement from consistent use. The PROSPECT trial examined 263 patients at 26 US clinical sites and evaluated the clinical safety and efficacy of using TAPS twice daily for three months [5]. The study included in-clinic assessments of modified versions of the Tremor Research Group’s Essential Tremor Rating Assessment Scale (TETRAS) [18] and patient-rated Bain & Findley Activities of Daily Living (BF-ADL) [19] scores, based on performance and task subsets, measured pre- and post-stimulation at enrollment and at 1-month and 3-month visits. The 8-item BF-ADL, including eating, drinking, writing, and self-care ADL tasks, was rated by patients after performing each task with in-office props (score range: 8 to 32). The 6-item TETRAS includes six performance items consisting of dominant hand tasks such as postural and action tremor in various positions, spiral drawing, writing, and a dot approximation task (score range: 0 to 24) and was rated by the study investigators [5].

This retrospective analysis evaluated underlying tremor improvement, as measured using pre-stimulation tremor assessments performed at baseline and compared to pre-stimulation assessments at prespecified trial visits, conducted after a washout period (mean ± SD: 16.2 ± 4.7 hours) following the last stimulation session. The responder rate for underlying tremor improvement was defined as the percentage of patients that improved at least one point on any individual task, based on pre-stimulation scores recorded at enrollment to scores at the 1-month or 3-month visit. This calculation was performed separately for BF-ADL tasks rated ≥3 at enrollment or for TETRAS tasks rated ≥2 at enrollment. The 1-point improvement aligns with Medicare coverage criteria only for BF-ADL [20], and was used as a responder definition in a prior publication [14]. The same improvement threshold was applied to TETRAS for consistency.

Patients in the PROSPECT trial demonstrated underlying tremor improvement from consistent use of TAPS. Of 192 patients with available data at both visits, pre-stimulation BF-ADL significantly improved by 2.0 points at one month and 2.7 points at three months compared to pre-simulation tremor at initial study enrollment (paired Wilcoxon signed-rank test, *V* = 11,221 and 13,520, respectively; *p* < 0.001). This improvement represented a shift in the cohort’s average score from 18.4 at enrollment to 15.8 at three months. TETRAS also showed significant improvements of 0.8 and 0.7 points at one and three months, respectively (*V* = 10,710 and 11,382; *p* < 0.001, mean scores at each time point shown in Figure 1A).

**Figure 1:**
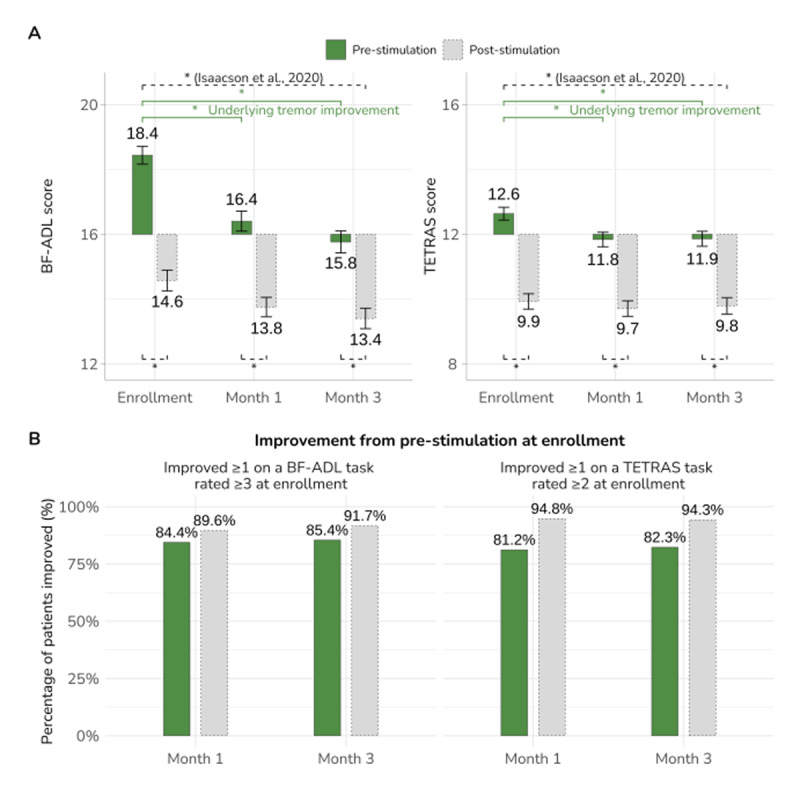
**(A)** Average BF-ADL dominant hand score (left, scale range 8 to 32) and TETRAS dominant hand score (right, scale range 0–24) are shown pre- and post-stimulation conducted at each in-clinic visit. Analysis of underlying tremor response (changes in BF-ADL and TETRAS scores) demonstrated that TAPS significantly improved underlying tremor severity, as measured by the pre-stimulation assessment, as well as providing tremor improvement post-stimulation as previously reported [5]. The origin of 16 (BF-ADL) and 12 (TETRAS) reflects an average score of 2 (i.e., ‘mild’) per task. Error bars represent ±1 SE and * indicates *p* < 0.001. **(B)** The percentage of patients who improved from pre-stimulation at enrollment are shown for both 1-month and 3-month visits. Analysis of the responder rate demonstrated that over 80% of patients demonstrated underlying tremor improvement as measured pre-stimulation and at least 90% improved post-stimulation.

The responder analysis showed that, at both the 1-month and 3-month visits, over 80% of patients in the PROSPECT trial had underlying tremor improvement based on pre-stimulation BF-ADL (binomial tests: 0.794, *p* < 0.001) and TETRAS (0.760, *p* < 0.001), analyzed separately (Figure 1B). To further assess whether these significant changes represent a clinically meaningful improvement, an additional analysis was performed on the BF-ADL data. At one month, 76.6% of patients had at least one task shift from a functionally-impaired state (i.e., Severe/Moderate, score of 3 to 4) at enrollment to a functionally-able state (i.e., Mild/No tremor, score of 1 to 2). This increased to 81.2% of patients at three months. Over 90% of patients also had acute or lasting improvement in tremor severity, as measured by post-stimulation BF-ADL and TETRAS, as previously reported (*p* < 0.001, Figure 1B).

Several limitations of this analysis warrant consideration. First, patients in the PROSPECT trial were instructed to use therapy twice daily [5]. Retrospective analysis revealed that patients completed 1.4 sessions per day on average, and even patients using therapy an average of only once per day experienced underlying tremor improvement (Table 1). Further research is needed to explore the effect of consistent TAPS on underlying tremor improvement with less frequent use. Second, this was an open-label study with no control arm. Therefore, improvements in underlying tremor severity may partly reflect learning or placebo effects or reduced stress and anxiety as patients become more familiar with the study team and procedures, given that stress and anxiety are known to exacerbate tremor. Therefore, a future, long-term, randomized controlled trial would be valuable to quantify the magnitude of this underlying improvement effect relative to a sham-controlled cohort. Third, while ET is considered a progressive disease for some patients, its course and rate of progression can be variable [21]. Longer studies are needed to determine whether consistent TAPS reduces the disease progression that might occur without intervention.

**Table 1:** Pre-Stimulation Tremor Improvement by TAPS Usage at 1-Month and 3-Month Visits.


FOLLOW-UP TIME	USAGE^a^	N	AVERAGE DAILY USAGE	TOTAL SCORE IMPROVEMENT (PRE-STIM)		RESPONDER RATE	
	
				BF-ADL	TETRAS	BF-ADL	TETRAS

1-Month	High	154	1.8 ± 0.0	2.2 ± 0.3	0.8 ± 0.2	85.1%	80.5%

	Low	38	1.0 ± 0.1	1.4 ± 0.6	0.8 ± 0.4	81.6%	84.2%

3-Month	High	140	1.8 ± 0.0	2.7 ± 0.3	1.0 ± 0.2	86.4%	83.6%

	Low	52	1.1 ± 0.0	2.7 ± 0.6	0.3 ± 0.4	82.7%	78.9%


Note: Descriptive statistics are reported in mean ± SE or percentages. ^a^High usage defined as use above the average number of daily sessions (i.e., 1.4 sessions per day) before the visit; low usage defined as below-average daily session use before the visit.

In conclusion, the retrospective analysis of the large essential tremor PROSPECT study, demonstrated underlying tremor improvement as measured by pre-stimulation assessments at one and three months, after consistent daily use of TAPS.

## Data Accessibility Statement

De-identified aggregate data relevant to this analysis may be available upon reasonable request.
